# Membrane-Active Metallopolymers: Repurposing and Rehabilitating Antibiotics to Gram-Negative Superbugs

**DOI:** 10.1002/adhm.202301764

**Published:** 2023-08-30

**Authors:** JiHyeon Hwang, Swagatam Barman, Ruixuan Gao, Xiaoming Yang, Andrea O’Malley, Prakash Nagarkatti, Mitzi Nagarkatti, Maksymilian Chruszcz, Chuanbing Tang

**Affiliations:** Department of Chemistry and Biochemistry University of South Carolina Columbia, SC 29208, USA; Department of Chemistry and Biochemistry University of South Carolina Columbia, SC 29208, USA; Department of Chemistry University of South Florida Tampa, FL 33620, USA; Department of Pathology Microbiology and Immunology University of South Carolina School of Medicine Columbia, SC 29209, USA; Department of Chemistry and Biochemistry University of South Carolina Columbia, SC 29208, USA; Department of Biochemistry and Molecular Biology Michigan State University East Lansing, MI 48824, USA; Department of Pathology Microbiology and Immunology University of South Carolina School of Medicine Columbia, SC 29209, USA; Department of Pathology Microbiology and Immunology University of South Carolina School of Medicine Columbia, SC 29209, USA; Department of Chemistry and Biochemistry University of South Carolina Columbia, SC 29208, USA; Department of Biochemistry and Molecular Biology Michigan State University East Lansing, MI 48824, USA; Department of Chemistry and Biochemistry University of South Carolina Columbia, SC 29208, USA

**Keywords:** adjuvants, biofilms, antimicrobial resistance, metallopolymers, stationary phase

## Abstract

Among multiple approaches to combating antimicrobial resistance, a combination therapy of existing antibiotics with bacterial membrane-perturbing agents is promising. A viable platform of metallopolymers as adjuvants in combination with traditional antibiotics is reported in this work to combat both planktonic and stationary cells of Gram-negative superbugs and their biofilms. Antibacterial efficacy, toxicity, antibiofilm activity, bacterial resistance propensity, and mechanisms of action of metallopolymer-antibiotic combinations are investigated. These metallopolymers exhibit 4-16-fold potentiation of antibiotics against Gram-negative bacteria with negligible toxicity toward mammalian cells. More importantly, the lead combinations (polymer-ceftazidime and polymer-rifampicin) eradicate preformed biofilms of MDR *E. coli* and *P. aeruginosa*, respectively. Further, *β*-lactamase inhibition, outer membrane permeabilization, and membrane depolarization demonstrate synergy of these adjuvants with different antibiotics. Moreover, the membrane-active metallopolymers enable the antibiotics to circumvent bacterial resistance development. Altogether, the results indicate that such non-antibiotic adjuvants bear the promise to revitalize the efficacy of existing antibiotics to tackle Gram-negative bacterial infections.

## 1. Introduction

Multidrug resistant (MDR) bacterial infections are among the most urgent threats that result in extensive socio-economic burden to the global healthcare.^[1–3]^ Alongside, the pipelines for drug discovery to fight the emergence of highly resistant pathogens have slowed down over the past few decades.^[4,5]^ Antibiotic resistance would remain as a significant issue even if new drug candidates emerge. Furthermore, the tendency of bacterial biofilm formation threatens the clinical settings owing to its severity ending up with no treatment by the conventional antibiotics.^[6,7]^ Therefore, the current situation demands novel therapeutics that can tackle drug resistance and bacterial biofilm-associated infections.

Inherited or acquired antibiotic resistance occurs through various mechanisms, including enzyme-enabled modification/degradation, decreased drug permeability, increased drug-efflux, etc.^[8–10]^ Enzymatic modification of the structures of antibiotics through hydrolysis is one of the bacterial survival mechanisms, rendering antibiotics ineffective.^[11]^ For example, *β*-lactamase-producing bacteria cleave the lactam ring in all penicillin and some of third-generation cephalosporins, including ceftazidime.^[11,12]^ Drug permeability in Gram-negative bacteria is more challenging than in Gram-positive bacteria due to the presence of an additional barrier of the outer membrane (OM).^[13,14]^ The extrusion of toxic substrates, including antibiotics, is another resistance mechanism by specific transport proteins called efflux pumps, which strongly depend on the membrane potential.^[15]^ On top of acquired drug resistance, drug-sensitive bacterial populations easily form biofilms, which, composed of extracellular polymeric matrices (EPS), serve as a strong barrier for antibiotic penetration.^[16]^

Combination therapy of multiple antibiotics or antibiotic(s) and adjuvants (non-antibiotic molecules) is one of the therapeutic approaches in the clinic to combat the complexities associated with MDR bacterial infections.^[17,18]^ This strategy increases the efficacy and reduces toxicity of existing antibiotics. Additionally, propensity of drug resistance by the bacterial populations can be minimized.^[19]^ A combination of antibiotics with adjuvants can suppress the development of resistance and achieve a synergistic effect.^[20]^ Adjuvants are typically classified into two groups based on their targets: Class 1 adjuvants that work on bacterial targets to inhibit antibiotic resistance and Class 2 adjuvants that enhance the efficacy of antibiotics to kill bacteria.^[8]^ Class 1 adjuvants are the only ones currently approved for clinical use, whereas Class 2 adjuvants are being explored in preclinical models.^[8]^ In an effort to resensitize MDR bacteria, several cationic non-antibiotic adjuvants have been reported to be effective.^[21–24]^

Antimicrobial macromolecules have been intensively explored due to their chemical robustness, stability, and long-term activity toward bacteria compared with small molecules.^[25,26]^ Moreover, antimicrobial polymers have been applied as surface coatings to prevent microbial accumulation in various products.^[27,28]^ Metal-based antimicrobial agents have gained considerable interest since the release of the clinically approved platinum-containing anticancer agents.^[29,30]^ However, metal-based antimicrobial macromolecules^[31–36]^ are less explored. Metal-containing polymers combines the processibility of an organic polymeric framework with functionalities from metal centers, which in turn tunes the properties of polymers.^[37–42]^

**Figure 1.**
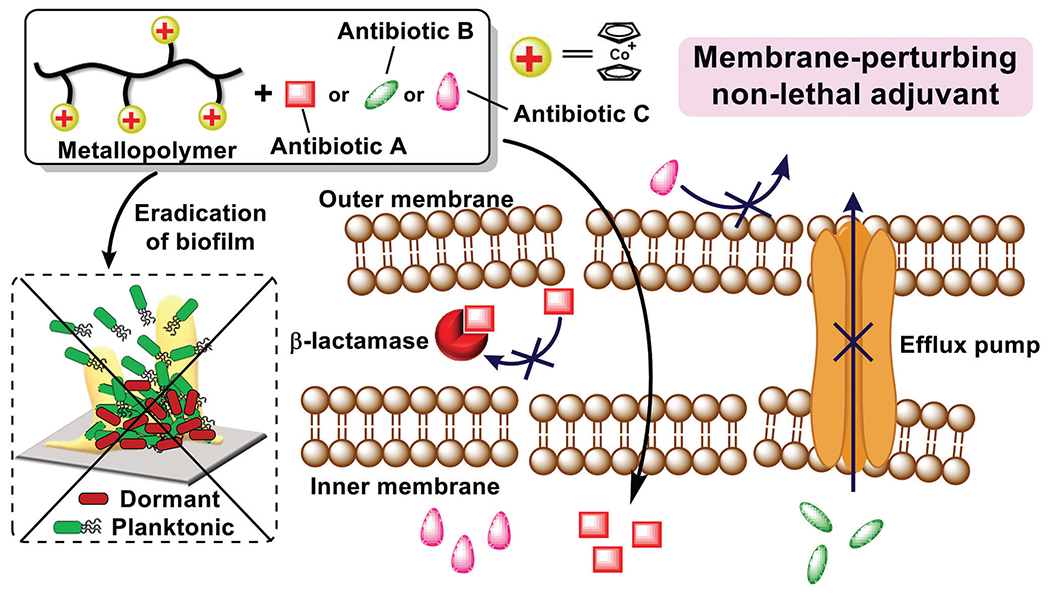
Illustration of antibiotic potentiation against Gram-negative bacteria by cationic metal-containing polymers via multiple mechanisms of action.

Herein, three different classes of antibiotics (ceftazidime, rifampicin, and minocycline) are combined with cationic cobaltocenium-containing polymers to revitalize their antimicrobial efficacy (Figure 1). The mechanisms of antibiotic potentiation were investigated via enzymatic hydrolysis, outer membrane permeabilization, membrane depolarization, and efflux pumps. Although bioconjugates of metallopolymers via electrostatic interactions have been reported,^[36,43–47]^ the current work investigated, for the first time, the effective utilization of the combinations of cationic metallopolymers with the existing drugs against both planktonic and stationary phase Gram-negative bacterial cells as well as bacterial biofilms. We focused on the synthetic polymer-antibiotic combinations exhibiting the ability to inhibit the growth of bacteria, high antibiofilm efficacy, and mechanisms of potentiation while maintaining low toxicity toward mammalian cells.

## 2. Results and Discussion

### 2.1. Synthesis of Polymer and Polymer-Antibiotic Combinations

**Scheme 1.**
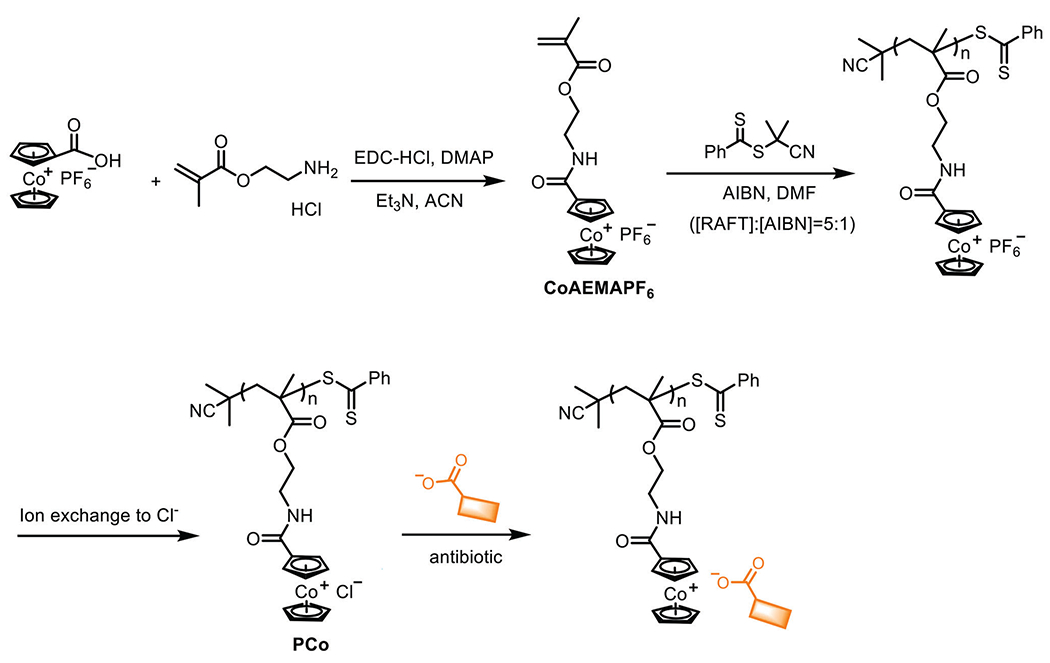
Synthesis of CoAEMAPF_6_, cobaltocenium-containing polymer (PCo, and conjugates with carboxylate-containing antibiotics.

Cobaltocenium, a cationic form of cobaltocene, is a unique cation that can induce electrostatic interaction with negatively charged bacterial membranes.^[36,43,44]^ Cobaltocenium-containing polymers were synthesized following reported protocols.^[43,48,49]^ Briefly, reversible-addition fragmentation transfer polymerization was carried out with 2-cobaltocenium amidoethyl methacrylate hexafluorophosphate (CoAEMAPF_6_) as a monomer, 2-cyano-2-propyl benzodithioate as a chain transfer agent, and 2,2′-azobisisobutyronitrile as an initiator (Scheme 1). The cobaltocenium-based polymer was highly soluble in water after ion exchange from PF_6_^−^ to Cl^−^ using tetrabutylammonium chloride. The number average molecular weight of the chloride-paired polymer (labeled as PCo) is 14 800 g mol^−1^ based on ^1^H-NMR spectroscopy and 27 200 g mol^−1^ based on gel permeation chromatography (GPC) with water as eluent (Figure S1, Supporting Information). It has been demonstrated that the molecular weight in the range of 5000–30 000 g mol^−1^ is optimal for interactions of polymers with bacterial cells.^[27,50,51]^ The carboxylate anion of *β*-lactam antibiotics (e.g., ceftazidime) formed a conjugate with cationic cobaltocenium moiety in the metallopolymer through anion exchange.

### 2.2. Antibacterial Activity of Polymer-Antibiotic Combinations

**Figure 2.**
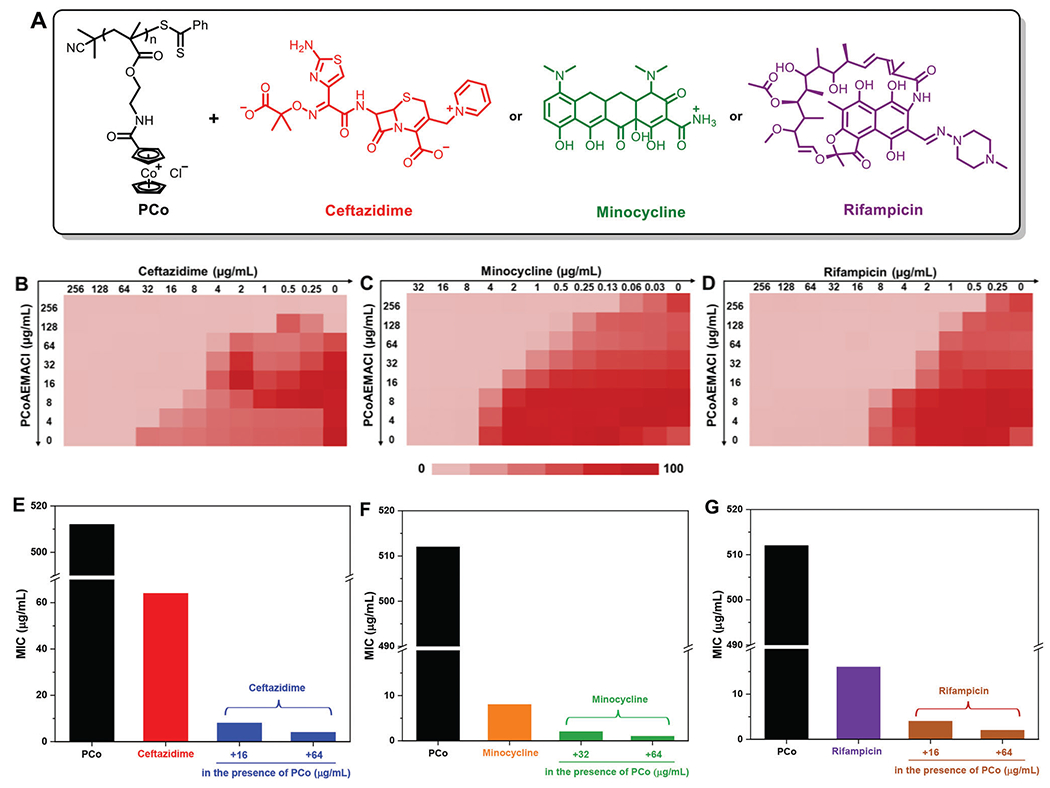
A) Combination of antibiotics (ceftazidime, minocycline, or rifampicin) with PCo. Checkerboard assay shows the combination efficacy of B) PCo-ceftazidime against MDR-*E. coli* (ATCC BAA-197) and C) PCo-minocycline and D) PCo-rifampicin against *P. aeruginosa* (ATCC-27853). Color gradient corresponds to turbidity at *λ* = 600 nm; Potentiating activity with E) ceftazidime against MDR-*E. coli*, F) minocycline and G) rifampicin against *P. aeruginosa* used for the PCo combination.

We studied polymer-antibiotic combinations, for which ceftazidime was first chosen as a model *β*-lactam antibiotic (Figure 2A). Ceftazidime is a third-generation cephalosporin antibiotic that is more effective against Gram-negative bacteria than Gram-positive bacteria. The antibacterial activity against different Gram-negative bacterial strains (*E. coli*, MDR *E. coli*, and *P. aeruginosa*) was expressed as minimum inhibitory concentration (MIC). PCo alone was not active and exhibited a MIC in the range of >512–256 μg mL^−1^ against all the tested pathogens (Table S1, Supporting Information). Similarly, ceftazidime alone was essentially ineffective against MDR *E. coli* (MIC = 64 μg mL^−1^), although it was active against drug-sensitive *E. coli* and *P. aeruginosa* with a MIC of 0.25 and 2 μg mL^−1^, respectively.

We then investigated the polymer-ceftazidime bioconjugates and discovered that the antibacterial activity against Gramnegative bacteria was highly dependent on the complex ratios of cobaltocenium cation to carboxylate anion. The potency of ceftazidime at variable dosages in combination with PCo was investigated using a checkerboard assay, in which both the concentrations of antibiotics and the polymer were controlled orthogonally (Figure 2B). The color mapping can determine the MICs of antibiotics with a specific concentration of the polymer. Further, other different classes of antibiotics (devoid of free carboxylate moieties) such as minocycline and rifampicin were also used in combination with the metallopolymer aiming to achieve a broad-spectrum antibacterial efficacy (Figure 2A,C,D). The latter combinations with neutral antibiotics do not alter the cationic charge density at the cobaltocenium moieties; thus, there should be no significant change on polymer solubility and aggregation.

In case of polymer-antibiotic combination approaches, we define synergistic interaction using a fractional inhibitory concentration index (FICI),^[52]^ which is determined as the sum of the MIC of each partner in combination divided by the MIC of the same partner before combination. FICI < 0.5 is considered as a synergistic combination, whereas FICI = 0.5–4 additive and FICI ≥ 4 antagonism. The PCo combinations with antibiotics are referred to as PCo-X, where X is the antibiotic (C: ceftazidime, M: minocycline, and R: rifampicin). For example, PCo64-R2 is 64 μg mL^−1^ of PCo in combination with 2 μg mL^−1^ of rifampicin. Although individual PCo was ineffective, most of its combinations with antibiotics improved the efficacy of the antibiotics (MIC) by 2- to 16-fold, displaying synergistic (S) or additive (A) effects (Figure 2 and Tables 1–3). The PCo-C combinations against *E. coli* revealed FICI ≥ 0.63, indicating only a 2-fold decrease of ceftazidime dosage. When PCo was tested in combination with rifampicin or minocycline (PCo-R and PCo-M) against *E. coli*, respective 8-fold and 16-fold reduction in MICs of minocycline (MIC_M,alone_ = 0.25 μg mL^−1^, MIC^M,combination^ = 0.03 μg mL^−1^) and rifampicin (MIC_R,alone_ = 4 μg mL^−1^, MIC_R,combination_ = 0.25 μg mL^−1^) were observed, demonstrating synergistic interactions and therefore antibiotic potentiation.

We further assessed the efficacy of the polymer and three antibiotics against MDR-*E. coli* and *P. aeruginosa* (Tables 1–3). The polymer (64 μg mL^−1^) resensitized ceftazidime against MDR-*E. coli* and substantially improved the MIC of the antibiotic by 16-fold (from 64 to 4 μg mL^−1^). However, PCo-R and PCo-M combinations exhibited additive interactions against MDR-*E. coli*. PCo-R combination was synergistic against *P. aeruginosa*. MIC of rifampicin was improved eightfold (MIC_R,combination_ = 2 μg mL^−1^) in presence of 64 μg mL^−1^ PCo, while additive response was observed at a lower concentration of PCo (8 μg mL^−1^). Similarly, the combinations of PCo at 64 μg mL^−1^ with ceftazidime and minocycline also demonstrated synergistic interactions against *P. aeruginosa*. This result indicated that the antibacterial efficacy of the antibiotics in combination is dependent on the concentration of PCo. Overall, the degree of synergistic effects was dependent on specific bacterial strains and antibiotics.

Based on the combination studies, PCo-C was selected against MDR-*E. coli* for further investigations described below, while PCo-M and PCo-R were chosen for use against *P. aeruginosa.*

### 2.3. Effects of PCo on *β*-Lactamase

**Figure 3.**
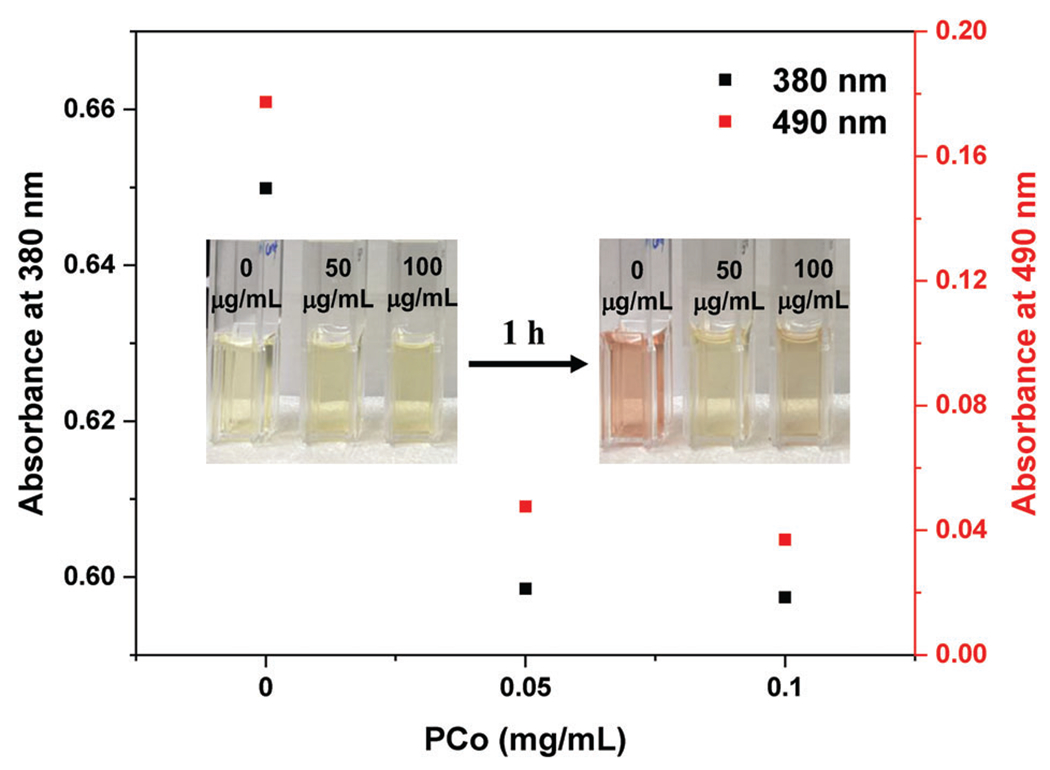
UV–vis absorption at 380 nm (black) and 490 nm (red) of nitrocefin solution with PCo and *β*-lactamase incubated for 0 and 1 h, respectively. Optical view of nitrocefin solution with different concentrations of PCo and *β*-lactamase at 0 and 1 h.

*β*-lactamases produced by both Gram-negative and Gram-positive bacteria can hydrolyze the lactam ring in *β*-lactam antibiotics, rendering them ineffective.^[53]^ In the case of Gram-negative bacteria, *β*-lactamases are among the primary causes of *β*-lactam antibiotic resistance. In order to assess the effects of PCo on *β*-lactamase activity, the enzyme was incubated with nitrocefin, typically used to indicate the existence and activity of a *β*-lactamase.^[54]^ We hypothesize that nitrocefin can be protected by a metallopolymer due to the ion-pairs of nitrocefin-metallopolymer conjugates between carboxylate anion in nitrocefin and cobaltocenium moieties of the metallopolymer.^[43]^ A concentration-dependent study was conducted by measuring UV-absorbance at 490 nm at different concentrations of PCo in the presence of nitrocefin and *β*-lactamase (Figure 3). As shown in Figure 3, the counter ion-exchange of Cl^−^ in the PCo led to ion-pairing interaction with the carboxylate anion in nitrocefin, reducing the absorption intensity of nitrocefin at 380 nm as opposed to the control (nitrocefin alone). Upon mixing *β*-lactamase in nitrocefin solution, color change from yellow to red indicated the hydrolysis of the *β*-lactam ring in nitrocefin with an absorption peak at 490 nm. The presence of PCo protected nitrocefin from hydrolysis by *β*-lactamase (up to 79% inhibition). Inhibition of *β*-lactamase hydrolysis via the metallopolymer is one of the possible mechanisms behind the synergy between ceftazidime and polymer.^[43,44]^

### 2.4. Outer Membrane Permeabilization

**Figure 4.**
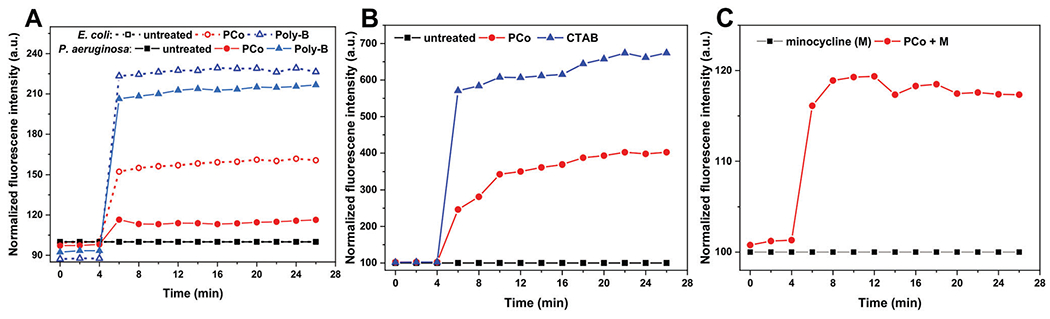
A) Outer membrane permeabilization of PCo (16 μgmL^−1^) and polymyxin-B (poly-B: 64 μg mL^−1^) against *E. coli* and *P. aeruginosa.* B) Membrane depolarization of *P. aeruginosa* by PCo (32 μg mL^−1^) and CTAB (64 μg mL^−1^). C) uptake of minocycline in the presence of PCo (32 μg mL^−1^) against *P. aeruginosa.* Minocycline alone was used at 128 μg mL^−1^.

Gram-negative bacteria comprise an outer membrane (OM), which consists of lipopolysaccharides (LPS), along with an inner membrane. For intrinsically drug-resistant bacteria, the OM serves as a barrier in preventing drug penetration.^[55]^ Perturbation of the OM can enhance the uptake of antibiotics, especially those with impaired influx problems, like rifampicin.^[43]^ Therefore, the OM perturbation ability of PCo was investigated against two different classes of Gram-negative (*E. coli* and *P. aeruginosa*) bacteria using an *N*-phenyl naphthylamine (NPN) dye through fluorescence assays (Figure 4A). NPN shows diminished fluorescence in an aqueous environment, while exhibiting strong fluorescence upon entering the hydrophobic region of a bacterial membrane with the perturbation of the bacterial OM. Polymyxin B (Poly-B), which interacts with the negatively charged LPS molecules of the OM through its hydrophobic fatty acid chain, was used as a positive control.^[56]^ Bacterial suspension devoid of any compound treatment was included as a control. An increase of NPN fluorescence intensity was observed when *E. coli* and *P. aeruginosa* were treated with 16 μg mL^−1^ PCo (only 1/16 and 1/32 MIC, respectively), confirming the OM perturbation. However, the extent of OM perturbation by the polymer was much less compared to Poly-B. Therefore, the polymer alone was nonlethal to the bacteria; instead, it facilitated the influx of the antibiotic rifampicin. In addition, the extent of the outer membrane permeability of PCo varied among different Gram-negative bacteria, e.g., a higher increment of fluorescence intensity against *E. coli* as compared to *P. aeruginosa*. The extent of membrane perturbation by PCo-R is consistent with that of potentiation ability, which did result in a twofold higher potentiation factor against *E. coli* (up to 16) as compared to *P. aeruginosa* (up to eight) (Table 3). These results demonstrated that Gram-negative bacterial OM was compromised by PCo, thereby enhancing the potency of rifampicin when used together with PCo.

### 2.5. Bacterial Membrane-Depolarization

In bacteria, the movement of protons across membrane generates an electrochemical gradient, known as the proton motive force (PMF). The PMF is composed of two parameters: electrical potential (ΔΨ) and the transmembrane proton gradient (ΔpH).^[57]^ ΔΨ and ΔpH components are interdependent; an increase in either component leads to a decrease of the other, maintaining a constant value of PMF.^[58]^ Minocycline belongs to the tetracycline class, and its uptake is known to be driven by ΔpH across the bacterial membrane.^[59]^ Hence, a compound dissipating the ΔΨ component of the PMF would result in an increased uptake of tetracycline antibiotics. In order to understand the synergistic interaction between minocycline and PCo, membrane-potential sensitive dye 3,3’-dipropylthiadicarbocyanine iodide (DiSC_3_) was used to monitor the cytoplasmic membrane depolarization by PCo in *P. aeruginosa.* This dye accumulates in the cytoplasmic membrane in response to ΔΨ and quenches its fluorescence. When ΔΨ component of the PMF is disturbed, or the membrane becomes permeabilized, the release of the dye into the solution results in an increase in fluorescence intensity. Cetyl trimethylammonium bromide (CTAB), a well-known toxic surfactant, was used as a positive control, showing a high membrane perturbation (Figure 4B). PCo at 32 μg mL^−1^ showed moderate membrane depolarization compared to the “gold standard,” CTAB, and this extent of ΔΨ dissipation was not lethal to the bacteria. Hence, we believe that PCo32-M2 and PCo64-M1 showed potentiation by reducing the MICs of minocycline to a therapeutic range (≤ 4 μg mL^−1^) (Table 2). Taken together, moderate membrane perturbation of PCo, compared to that of CTAB, was enough to have potentiation effects on minocycline, reducing its dosage against Gram-negative bacteria.

### 2.6. Minocycline Uptake

To further validate the effects of PCo on the interplay between ΔΨ and ΔpH, the influx of minocycline toward *P. aeruginosa* was monitored. The uptake of minocycline was measured through the increment in its fluorescence intensity as it enters inside the bacterial cell membranes.^[60]^ In the presence of PCo (32 μg mL^−1^), an increased uptake was observed compared to minocycline alone (Figure 4C). Since PCo dissipated the ΔΨ component of the PMF, bacterial cells are expected to compensate the overall PMF by increasing the ΔpH component. Therefore, we believe that the membrane active PCo increased the uptake of minocycline into the bacteria.

### 2.7. Toxicity of PCo and Its Combinations with Antibiotics

**Figure 5.**
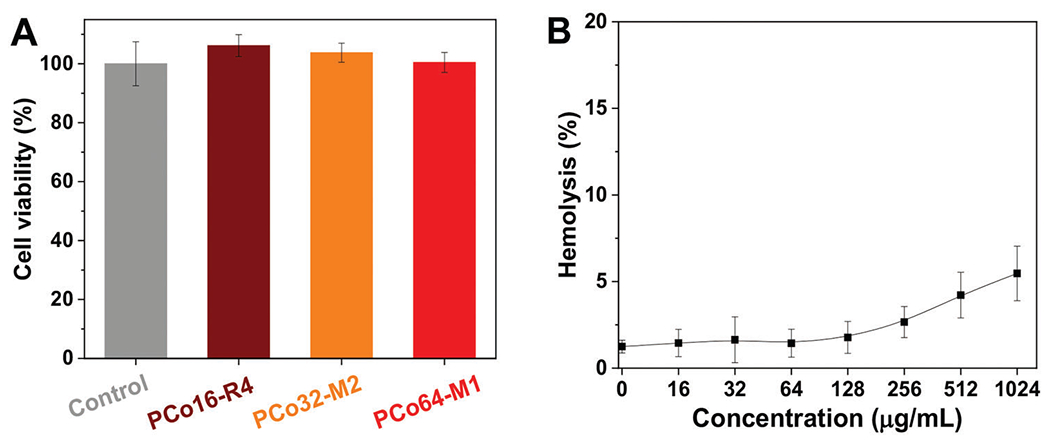
A) Cytotoxicity of HEK293T cells by PCo, PCo16-R4, PCo32-M2, and PCo64-M1. B) Hemolytic activities of PCo at increasing concentrations (16, 32, 64, 128, 256, 512, and 1024 μg mL^−1^) against mouse red blood cells. *N* = 4 for all studies.

The cytotoxicity effects of PCo and PCo-antibiotic combinations on human embryonic kidney (HEK293T) cell lines were evaluated by quantifying the release of lactate dehydrogenase (LDH) (Figure 5A; and Figure S2, Supporting Information). The IC_50_ (concentration of test drug required for 50% cytotoxicity of human embryonic kidney cells) was determined as >1024 μg mL^−1^ for PCo. We further assessed three potent combinations with concentrations that displayed synergistic interaction, PCo16-R4, PCo32-M2, and PCo64-M1, and these combinations led to almost 0% cytotoxicity. The activity of PCo on mouse red blood cells (RBCs) was also studied by hemolysis. PCo exhibited nontoxic nature at various concentrations, and the HC_50_ (concentration of test drug required for 50% lysis of RBCs) was found to be >1024 μg mL^−1^ (Figure 5B). This hemolytic data displayed nontoxicity to red blood cells.

### 2.8. Bactericidal Kinetics of Combinations against Planktonic and Stationary Cells of Gram-Negative Bacteria

Bacteria are at a stationary phase when the concentration of nutrients is too low for growth, so the biological processes of bacteria slow down. Antibiotics that target planktonic bacteria (metabolically active) is often ineffective in treating metabolically inactive bacteria (stationary phase) because of the different metabolic state of the bacteria.^[61]^ Therefore, the kinetics of killing for the polymer-antibiotic combinations were determined against both the planktonic and stationary cells of two Gram-negative bacteria, MDR *E. coli* and *P. aeruginosa*.

**Figure 6.**
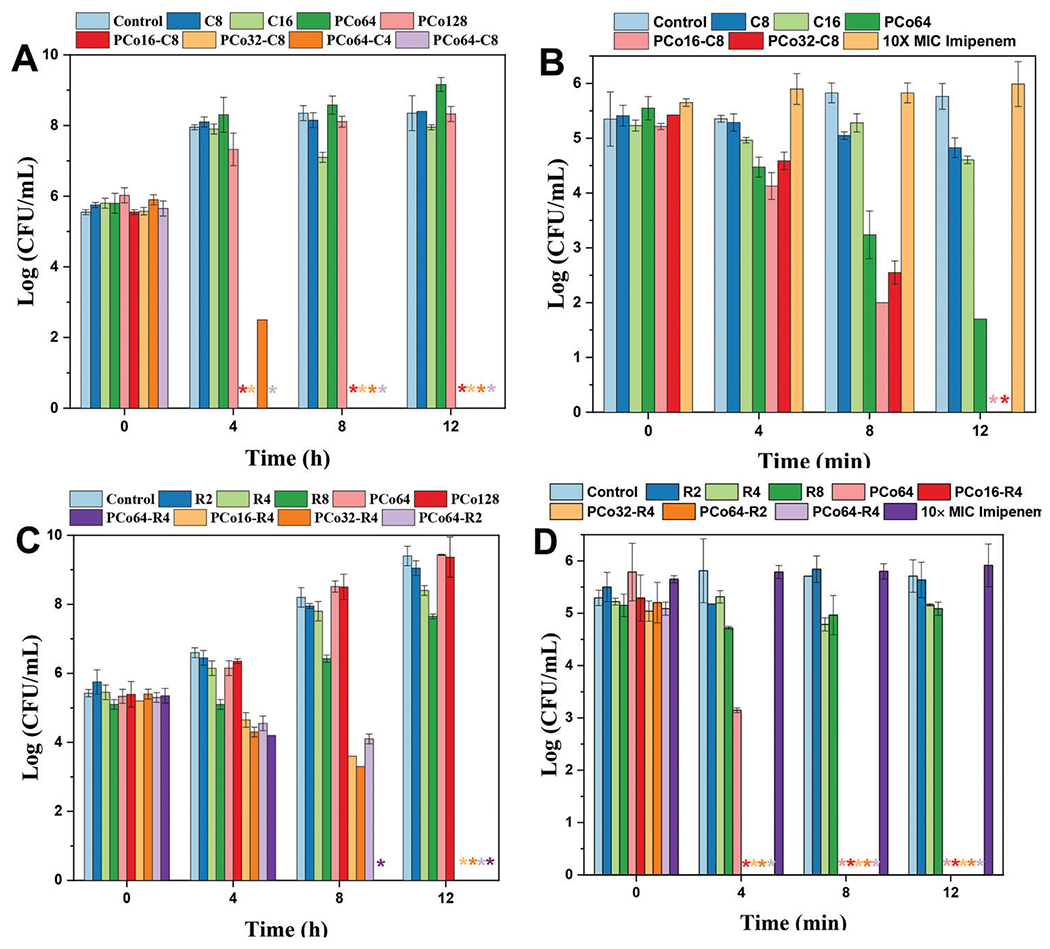
Time-kill kinetics: PCo, ceftazidime, and PCo-ceftazidime (PCo-C) combinations against A) MDR-*E. coli* and B) stationary cells of MDR-*E. coli*; PCo, rifampicin, and PCo-rifampicin (PCo-R) combinations against C) *P. aeruginosa* and D) stationary cells of *P. aeruginosa*. 2.5 and 10 μg mL^−1^ of imipenem (10 × MIC) was used as a control against stationary cells of MDR-*E. coli* and *P. aeruginosa*, respectively. *N* = 3 for all studies. The asterisk (*) indicates <50 CFU.

The PCo64-C4 combination completely eliminated (5.9 Log reduction) the growing planktonic MDR *E. coli* cells within 8 h, whereas PCo alone (64 and 128 μg mL^−1^) and ceftazidime alone (8 and 16 μg mL^−1^) did not show any noticeable bactericidal effects within 12 h, similar to the control (Figure 6A). At a higher concentration of ceftazidime in PCo64-C8, faster bacterial elimination (5.7 Log reduction) was observed within 4 h. We also varied the concentrations of PCo from 64 to 32 to 16 μg mL^−1^ in the presence of ceftazidime at 8 μg mL^−1^. At a lower concentration of polymer (PCo32-C8 and PCo16-C8), the complete killing (5.6 Log reduction) of the planktonic phase of MDR-*E. coli* was observed within 4 h. These results demonstrated faster bactericidal kinetics of the PCo-C combinations for the planktonic phase of MDR *E. coli* cells. Time-kill kinetics against the planktonic cells of *P. aeruginosa* were also evaluated with the PCo-R and the PCo-M combinations. The PCo-R combinations (PCo16-R4 and PCo32-R4) showed the bactericidal nature of the combination, eliminating the growing *P. aeruginosa* cells completely (5.2 and 5.4 Log reduction, respectively) within 12 h (Figure 6C). The combination of lower concentration of rifampicin (2 μg mL^−1^) and a higher concentration of PCo (64 μg mL^−1^) was equally effective. The PCo64-R4 combination consisting of a twofold higher concentration of rifampicin displayed a faster bacterial killing kinetics within 8 h. However, individual PCo (64 and 128 μg mL^−1^) and rifampicin (2, 4, and 8 μg mL^−1^) did not show any lethal effects on the planktonic bacteria. Next, we tested the efficacy of the PCo-M combinations against the planktonic cells of *P. aeruginosa* cells. All combinations (PCo32-M2, PCo64-M1, PCo64-M2, and PCo64-M4) exhibited significant reduction of bacterial burden (≈2.5 Log CFU mL^−1^ reduction) within 12 h (Figure S3A, Supporting Information). However, minocycline (2 and 4 μg mL^−1^) alone did not show any bacterial killing within 12 h, similar to the control. The results all together suggested a moderate to rapid bactericidal nature of the combinations.

Conventional antibiotics are more refractory to killing stationary phase bacteria due to decreasing cellular processes where antibiotics target.^[62]^ However, PCo-antibiotics combinations and PCo (64 μg mL^−1^) alone were able to kill stationary phase cells of *P. aeruginosa* completely (≈5–6 Log CMF mL^−1^ reduction) within 4 and 8 h, respectively (Figure 6D; and Figure S3B, Supporting Information). Although the PCo-antibiotic combinations were found to kill stationary phase cells more rapidly than planktonic cells, it should be noted that the killing kinetics of planktonic and stationary phase bacteria were performed in the nutrient-rich Cation-adjusted Mueller–Hinton broth (CAMHB) and nutrient-deficient phosphate buffered saline (PBS), respectively. Similarly, PCo16-C8 and PCo32-C8 completely killed (≈5–6 Log reduction) stationary phase cells of MDR *E. coli* within 12 h and PCo (64 μg mL^−1^) alone reached ≈3.8 Log reduction (Figure 6B, Supporting Information). The efficacy of the metallopolymer-antibiotic combinations was compared with imipenem. 2.5 and 10 μg mL^−1^ of imipenem (10 × MIC for MDR *E. coli* and *P. aeruginosa*, respectively) failed to kill both stationary cells of MDR *E. coli* and *P. aeruginosa* bacteria within 12 h, whereas it was active against their planktonic bacterial cells.

### 2.9. Biofilm Disruption

**Figure 7.**
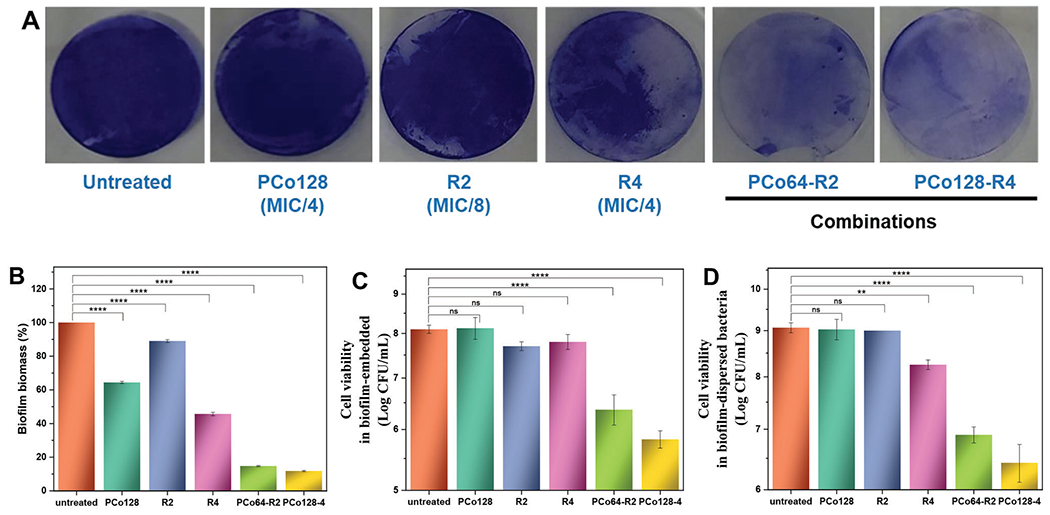
Biofilm disruption by PCo, rifampicin, and PCo-rifampicin (PCo-R) combinations for *P. aeruginosa*: A) visualization by CV staining. B) amount of biomass (%) in biofilms (*N* = 4); quantification of cell viability of C) biofilm-embedded bacteria (*N* = 3) and D) biofilm-dispersed bacteria (*N* = 3). ns, *, **, ***, and **** indicate *p* > 0.1, *p* < 0.1, *p* < 0.01, *p* < 0.001, and *p* < 0.0001, respectively, as determined by one-way ANOVA with Dunnett’s multiple comparison test (ns stands for nonsignificant).

According to the US National Institutes of Health, more than 80% of infections are derived from biofilm-related infections.^[63]^ Biofilms are rigid assemblies or aggregates of bacteria surrounded by a self-produced EPS.^[64,65]^ Moreover, bacteria in biofilms are 1000 times more antibiotic-resistant than planktonic bacteria.^[6]^ Therefore, conventional antibiotic therapeutics often fail in treating biofilm infections safeguarded with dormant species. Having established the faster bactericidal nature of PCo-antibiotic combinations against planktonic bacterial cells, we further assessed whether the PCo-rifampicin (PCo-R) and PCo-ceftazidime (PCo-C) combinations could have a synergistic activity against biofilms of *P. aeruginosa* and of MDR *E. coli*, respectively. Bacterial biofilms of *P. aeruginosa* and MDR *E. coli* were grown over glass coverslips, and the potency of the combinations was compared with individual antibiotics (rifampicin and ceftazidime) and PCo against the biofilms. To visualize the extent of biofilm disruption, crystal violet (CV) staining was performed. The staining clearly exhibited that the combinations (PCo-R and PCo-C) disrupted preformed biofilm biomass compared to the untreated, antibiotic alone, and polymer alone, as shown in Figure 7A; and Figure S4A (Supporting Information). PCo at a concentration of 128 μg mL^−1^ (MICs: >512 and 512 μg mL^−1^ against *P. aeruginosa* and MDR *E. coli*, respectively) showed ≈36% and 30% reduction in *P. aeruginosa* and MDR *E. coli* biofilm biomass, respectively (Figure 7B; and Figure S4B, Supporting Information). PCo64-R2 and PCo128-R4 reduced ≈85% and ≈88% biofilm biomass of *P. aeruginosa*, respectively, while rifampicin alone reduced ≈11% and ≈54% biofilm biomass at 2 and 4 μg mL^−1^, respectively. PCo64-C4 and PCo128-C8 displayed ≈70% and 80% reduction of biofilm biomass of MDR-*E. coli*, whereas ceftazidime rather increased the biomass. The results showed that PCo, when combined with rifampicin and ceftazidime, significantly eradicated the preformed biofilm biomass compared to the untreated sample.

Further, we quantified the biofilm disruption efficacy of the combinations by evaluating the cell viability of biofilm embedded and biofilm dispersed bacteria. The results suggested that PCo64-R2 and PCo128-R4 displayed significant reduction (1.8 and 2.3 Log CFU mL^−1^, respectively) of cell viability of biofilm embedded bacteria in the preformed biofilms of *P. aeruginosa* compared to untreated (control), antibiotic alone (0.3 and 0.4 Log CMF mL^−1^ at concentrations of 2 and 4 μg mL^−1^ of rifampicin, respectively), and polymer alone (no reduction) (Figure 7C). In addition, the results of the cell viability assessment of biofilm-embedded MDR *E. coli* treated with PCo64-C4 and PCo128-C8 exhibited 98% (1.9 Log CFU mL^−1^) and 99% (2 Log CFU mL^−1^) killing, respectively (Figure S4C, Supporting Information). We further observed that PCo64-R2 and PCo128-R4 reduced cell viability (2.2 and 2.6 Log CMF mL^−1^) even in the dispersed bacteria from the preformed biofilm biomass (Figure 7D). We also observed 97% (1.6 Log) killing of biofilm-dispersed MDR *E. coli* by both PCo64-C4 and PCo128-C8 (Figure S4D, Supporting Information). Therefore, the combinations were efficacious to prevent further biofilm colonization at the distal site of infection through biofilm dispersed cells, a virulence factor. Since the metallopolymer is cationic in nature, it may interact with various negatively charged components of biofilm matrix (EPS), which include DNAs, proteins, carbohydrates, etc. This further facilitated the entry of antibiotics to circumvent the EPS barrier and resulted in a detrimental effect on the biofilm embedded bacterial populations. Taken together, synergistic polymer-antibiotic combinations can be promising antibacterial agents and antibiofilm agents.

### 2.10. Drug Resistance

**Figure 8.**
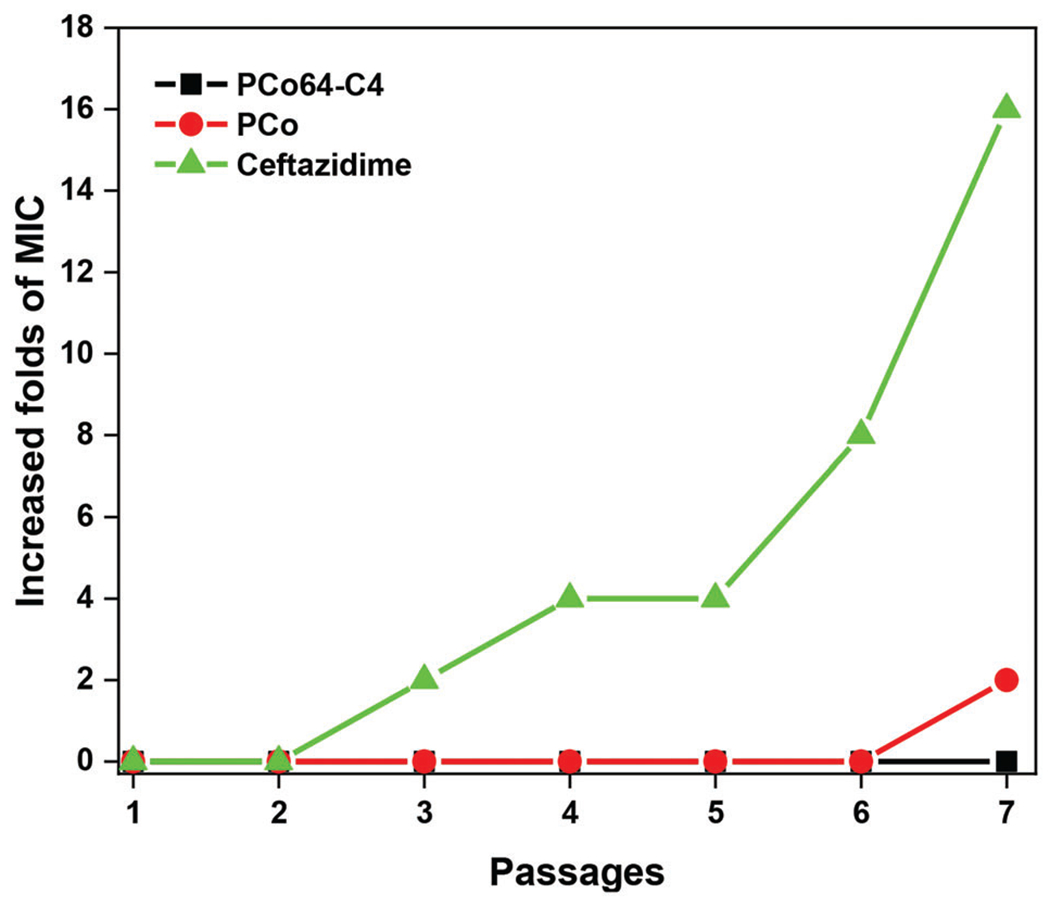
Propensity of resistance development of PCo64-C4, PCo (512 μg mL^−1^), and ceftazidime (64 μg mL^−1^) against MDR-*E. coli.*

The continued use of antibiotics frequently results in the development of drug resistance by bacteria through modifying defense elements. Therefore, it is critical to investigate the prolonged activity of a new antibacterial agent against bacteria. We assessed the MIC of ceftazidime in presence of the metallopolymer (64 μg mL^−1^) against MDR *E. coli* upon multiple bacterial exposures or passages. It was aimed to investigate whether the membrane-active metallopolymer can hold the bacterial resistance development against the antibiotic or not. Figure 8 shows that the MIC value of PCo (512 μg mL^−1^) alone increased only two-fold after seven passages. The MIC of ceftazidime in the presence of PCo (64 μg mL^−1^) did not alter, whereas a 16-fold increase of MIC was observed in the case of ceftazidime alone. This result indicated that the polymer PCo made ceftazidime immune to bacterial resistance development, and thus the combination of PCo-ceftazidime can be a promising candidate for an antibacterial therapeutic agent with long-lasting efficacy. The membrane-active nature of the metallopolymer was attributed to no detectable antibiotic resistance.

## 3. Conclusions

In conclusion, the combination of antibiotics with cobaltocenium-containing polymers enhanced the antibacterial activity and revitalized the activity of the antibiotics through multitargeting approaches. The synergetic interaction through combination extends the lifetime of traditional drugs, reducing the propensity of resistance. Combined therapies acted simultaneously in killing microbes, thereby showing a more significant effect than equivalent doses of individual antimicrobial agents.

PCo interacts with the negatively charged bacterial membrane, favoring the action of the antibiotics. Most importantly, the lead combination (PCo-R and PCo-C) displayed significant biofilm disruption and lethal effects on both biofilms embedded and dispersed bacteria, which are metabolically distinct in nature. Overall, this work could pave a new pathway in understanding and improving the performance of cationic metallopolymers as a non-antibiotic adjuvant in combination with existing antibiotics. Similar to many of reported antimicrobial polymers that may not be degraded in the body, which may circumvent renal filtration and increase toxicity to patients, further research is required to develop biodegradable polymers as antimicrobial agents.

## 4. Experimental Section

### General Methods and Materials:

All reagents were used as received unless otherwise stated. Cobaltocenium-containing methacrylate polymers with hexafluorophosphate anion (poly(2-(methacrylolyoxy)ethyl cobaltoceniumcarboxylate hexafluorophosphate)) and its chloride paired cobaltocenium-containing polymers were synthesized according to the earlier reports^[49,66]^ (*M*_n_ = 14 800 g mol^−1^ (^1^H NMR) and 27 200 g mol^−1^ (GPC), *M*_w_/*M*_n_ = 1.1). Nitrocefin was purchased from TOKU-E and used as received. Ceftazidime monohydrate, minocycline hydrochloride, and rifampicin were purchased from VWR and used as received.

### Characterization:

^1^H NMR (400 MHz) spectra were recorded on a Varian Mercury 300 spectrometer using D_2_O as solvent. Gel permeation chromatography (GPC) was performed in 0.1 m NaCl with 0.1% trifluoroacetic acid/acetonitrile (80/20 v/v) at a flow rate of 1.0 mL min^−1^ at 35 °C on 1260 Infinity II system equipped with PSS Novema Max columns and a refractive index (RI) detector, and narrow dispersed pullulan polymer was employed for molecular weight calibration. UV–vis spectra and optical density (OD) measurements were recorded on SpectraMax M5 Multimode Microplate Reader, Molecular Devices.

### Bacterial Culture Condition:

*Escherichia coli* (*E. coli*, ATCC-25922), *Pseudomonas aeruginosa* (*P. aeruginosa*, ATCC-27853), and *Escherichia coli* (Migula) Castellani and Chalmers (MDR *E. coli*, ATCC BAA-197) were purchased from ATCC. Bacteria were streaked onto tryptic soy agar and incubated at 37 °C for 16 h. Then, a single colony was inoculated into 4 mL of fresh tryptic soy broth (TSB) and incubated at 37 °C for 6 h, while under shaking at 190 rpm. For MDR *E. coli*, TSB containing 10 μg mL^−1^ of ceftazidime was used. All bacteria were grown to a mid-log phase for further use.

### Minimum Inhibitory Concentration Assay:

50 μL of an aqueous solution of compounds with different concentrations was added to 96-well plates. Then, 50 μL of bacterial solution with a concentration of 10^6^ CFU mL^−1^ was added to each well. The plate was incubated at 37 °C for 18 h, and the absorbance (OD_600_) was measured. CAMHB without polymers, antibiotics, and bacteria was used as the negative control. The bacteria solution without polymers and drug was used as the positive control.

Antibacterial assay for polymers-antibiotics combinations: 25 μL of chloride (Cl^−^)-paired cobaltocenium-containing polymer, PCo, was serially diluted twofold in 96-well plates along the column. Then, 25 μL of antibiotic solution of the twofold serially diluted solution was added into the wells. 50 μL of bacterial solution (10^6^ CFU mL^−1^) in CAMHB was added to the well with 25 μLofpolymerand 25 μL of antibiotic solution. After 18 h incubation at 37 °C, OD values of the plates were recorded at 600 nm.

### Production of Recombinant β-Lactamase and Effects of PCo on β-Lactamase Activity:

DNA encoding for the penicillin-hydrolyzing class A *β*-lactamase from *Staphylococcus aureus* (including an N-terminal hexahistidine tag and a tobacco etch virus (TEV) cleavage site, followed by *β*-lactamase residues 25–281) was inserted into the pJ411 vector for recombinant expression with *Escherichia coli*. The DNA was transformed into DH5*α E. coli* cells, the plasmid was purified with the GeneJET Plasmid Miniprep kit (Thermo Fisher Scientific, Waltham, MA), and the plasmid was transformed into BL-21(DE3) *E. coli* cells. 5 mL lysogeny broth (LB) cultures were inoculated with transformed cells and 50 μg mL^−1^ kanamycin and were grown overnight at 37 °C under shaking at 200 RPM. The 5 mL cultures were used to inoculate 1 L LB cultures, which were grown at 37 °C under shaking at 200 RPM until an optical density (OD_600_) of 0.6–0.8. After induction with 400 μm IPTG, the cells were grown overnight at 16 °C and shaking at 150 RPM.

1 L cultures were centrifuged at 9000 x *g* and resulting pellets were solubilized in lysis buffer (20 mm Na_2_HPO_4_ pH 6.5, 500 mm NaCl, 10 mm imidazole, 2% glycerol). The supernatant was sonicated and centrifuged at 35 000 x *g*. The resulting supernatant was poured into a 12 × 1.5 cm Bio-Rad column with 5 mL of nickel resin previously equilibrated with wash buffer (20 mm Na_2_HPO_4_ pH 6.5, 500 mm NaCl, 30 mm imidazole, 2% glycerol). Supernatant and wash buffer were alternated until the final wash step, and the protein was eluted from the column with elution buffer (20 mm Na_2_HPO_4_ pH 7.5, 500 mm NaCl, 300 mm imidazole, 2% glycerol).

The purification tags were cleaved with TEV at a 1:50 (TEV: *β*-lactamase) ratio, and cleaved protein was used for further experiments. The protein underwent secondary purification with size-exclusion chromatography with phosphate buffer (20 mm Na_2_HPO_4_ pH 6.5, 150 mm NaCl). Fractions corresponding to clean *β*-lactamase were collected, with purity verified with 12% SDS-PAGE. The protein was diluted with 20 mm Na_2_HPO_4_ pH 6.5 buffer prior to activity analyses.

The amount of this enzyme in solution was 0.8 μg mL^−1^ (determined by Bio-Rad Protein Assay). 50 μL of nitrocefin (1 mg mL^−1^ in DMSO) was added into PCo and diluted to 1 mL using 20 mm Na_2_HPO_4_ pH 6.5 buffer. Then, 100 μL of the purified *β*-lactamase was added to the above solution. The final concentration of each polymer was set at 0.05, 0.1, 0.4, 0.8, and 1.6 mg mL^−1^. A solution without polymers was prepared as a control. *β*-lactamase activity was measured by absorbance of hydrolyzed nitrocefin (490 nm) at different time intervals. Inhibition of *β*-lactamase activity was calculated as

(1) $$\text{inhibition}\;(\%)=\left(1-\frac{{\text{Abs}}_{490}{(t=1)-\text{Abs}}_{490}(t=0)}{{\text{Abs}}_{490}{\left(t=1\right)}_{c}{-\text{Abs}}_{490}{\left(t=0\right)}_{c}}\right)\times 100$$

Abs_490_ (*t* = 0) indicates the initial Abs_490_ value and Abs_490_ (*t* = 1) is the Abs_490_ value after 1 h incubation of bacterial cell with PCo. Abs_490_ (*t* = 0)_*c*_ is the initial Abs_490_ value and Abs_490_ (*t* = 1)_*c*_ is the Abs_490_ value after 1 h incubation of bacterial cells.

### Outer Membrane Permeabilization Assay:

This assay was performed with PCo and Poly-B (64 μg mL^−1^) against MDR *E. coli* and *P. aeruginosa*. Poly-B, the last-resort Gram-negative antibiotic, was used to compare the result. Briefly, bacterial cells (10^8^ CFU mL^−1^) were collected by centrifugation at 10 000 x *g* for 5 min and washed with a 1:1 ratio of 5 mm glucose and 5 mm 4-(2-hydroxyethyl)-1-piperazineethanesulfonic acid (HEPES) buffer (pH 7.4). Bacterial cells were resuspended in the same mixture of buffer solution. Next, the suspension was mixed with 10 *μ*M of NPN. 190 μL of NPN-containing bacterial suspension was transferred to a Corning 96-well black plate with a clear bottom. The fluorescence of NPN was monitored for 4 min with an excitation wavelength at 350 nm and an emission wavelength at 420 nm. 10 μL of PCo was added to every well containing dye-loaded bacterial suspension to attain the final concentration of 16 μg mL^−1^. Fluorescence was recorded for the next 22 min. Poly-B, with the final concentration of 64 μg mL^−1^, was used as a positive control.

### Cytoplasmic Membrane Depolarization Assay:

*P. aeruginosa* (10^8^ CFU mL^−1^) was collected by centrifugation at 10 000 x *g* for 5 min and washed with a 1:1 ratio of 5 mm glucose and 5 mm HEPES buffer. Bacterial cells were resuspended in a 1:1:1 ratio of 5 mm glucose, 5 mm HEPES buffer, and 100 mm KCl with 0.2 mm ethylenediaminetetraacetic acid (EDTA). This assay was performed in a Corning 96-well black plate with a clear bottom containing 190 μL of bacterial suspension and 2 μm DiSC_3_. The plate was kept at room temperature for 1 h, and the fluorescence of the suspension was monitored for 4 min with an excitation wavelength at 622 nm and an emission wavelength at 670 nm. 10 μL of PCo was added into each well to have the final concentration of 16 μg mL^−1^. The fluorescence was read continuously for the next 22 min at an interval of 2 min. The negative control was untreated *P. aeruginosa*. CTAB with the final concentration of 64 μg mL^−1^ was used as positive control giving 100% permeabilization.

### Tetracycline Uptake Assay:

*P. aeruginosa* (10^8^ CFU mL^−1^) was collected by centrifugation at 10 000 x g for 5 min and washed with 10 mm HEPES buffer. Bacterial cells were resuspended in the same buffer and minocycline was added to the bacterial suspension. 190 μL of this mixture were then transferred to a Corning 96-well black plate with a clear bottom, and the fluorescence was measured for an initial 4 min with an excitation wavelength at 405 nm and an emission wavelength at 535 nm. 10 μL of PCo was mixed with the solution of bacteria and minocycline. The final concentrations of PCo and minocycline were 32 and 128 μg mL^−1^, respectively. Fluorescence was measured for the next 22 min with an interval of 2 min. 10 μL of sterile DI water was used as a negative control.

### LDH Toxicity Assay:

HEK293T cells (Dulbecco’s Modified Eagle Medium with 10% fetal bovine serum (FBS)) were seeded in a 96-wall plate 1 day prior to experiments. At the time of testing, cell confluency was between 50% and 80%. The medium was changed to DEMM w/ 5% PBS (the total volume is 150 μL). Polymer-antibiotic combinations with different concentrations were added to the wells and incubated at 37 °C for 8 h. Cells incubated with lysis buffer from the kit (BioLegend LDH-Cytox Assay kit, cat#426 401) were used as maximal toxicity controls. After incubation, 100 μL supernatant from each well was transferred to a new 96-well-reading plate. To a 96-well plate containing supernatant, 100 μL of LDH assay working solution was added according to the instruction provided. The absorbance was measured at 490 nm. Wells with medium only served as background controls. Relative toxicity calculation

(2) (Sample OD−background)/(Max Lysis OD−background)

### Hemolytic Activity:

All experimental procedures performed were approved by the University of South Carolina Institutional Care and Use Committee (IACUC) according to the guidelines set by the Care and Use of Laboratory Animals of the National Research Council under AUP#2508. Blood was collected from mice in heparinized tubes and washed with PBS+10% FBS. PCo was prepared in PBS at different concentrations and added to the blood in a 96-well plate (150 μL). PBS with 0.2% Triton-X100 served as a maximal lysis control. The samples were incubated for 1 h at 37 °C followed by centrifugation for 10 min at 4000 x g. Supernatants (100 μL) were transferred to a 96-well plate, and OD was measured at 450 nm. PBS+10% FBS was used for background reading. The hemolysis rate was calculated as

(3) (Sample OD−background)/(Max Lysis OD−background)

### Time-Kill Kinetics against Planktonic Bacteria:

PCo, antibiotic, and the polymer-antibiotic combinations were tested using the method mentioned in the MIC test. At each time (0, 4, 8, and 12 h), the solution was diluted 10, 10^2^, 10^3^, 10^4^, 10^5^, 10^6^, and 10^7^-fold and then 20 μL was spread on the TSB agar plate. The result was presented on a logarithmic scale.

### Time-Kill Kinetics Against Stationary Phase Bacteria:

*Time-kill Kinetics against Stationary Phase Bacteria* To grow the stationary phase bacteria, 3 μL of bacteria was inoculated in 3 mL of TSB and incubated for 6 h at 37 °C, while under shaking at 190 rpm. Then 5 μL of bacterial solution was inoculated in 5 mL of TSB and incubated for 16 h at 37 °C, while under shaking at 190 rpm. Next, the stationary phase bacterial solution was centrifuged at 10 000 x g for 5 min and the supernatant was removed. The pellet was resuspended in 5 mL of 1× PBS and centrifuged at 3500 rpm for 5 min. The supernatant was removed, and the pellet was diluted in 1× PBS for further use. PCo, antibiotic, and the polymer-antibiotic combinations were tested using the method mentioned in the MIC test. At each time (0, 4, 8, and 12 h), the solution was diluted 10, 10^2^, 10^3^, 10^4^, and 10^5^-fold and then 20 μL was spread on the TSB agar plate. The result was presented on a logarithmic scale.

### Biofilm Disruption Assay:

*P. aeruginosa* and MDR *E. coli* biofilms were grown over glass coverslips using a nutrient broth with 1% w/v glucose and 1% w/v NaCl supplements. 6 h grown culture of bacteria (2 mL) was added to each well of a 6-well plate containing sterilized coverslips. The plate was incubated at 30 °C for 72 h. After 3 days of incubation, each biofilm-containing coverslip was washed gently with 1× PBS (pH 7.4) to remove the suspension and transferred to a new well plate. 2 mL of rifampicin (2 and 4 mg mL^−1^), ceftazidime (4 and 8 mg mL^−1^), PCo (128 mg mL^−1^), or the polymer-antibiotic combinations with different final concentrations was added to wells containing biofilm-coated coverslips and incubated for 24 h. Biofilm media alone was used as a negative control. Subsequently, the supernatant was discarded, and the biofilms treated with compounds were washed with 1× PBS and transferred to a new 6-well plate. 2 mL of methanol was added to the biofilm-containing coverslips and further incubated for 20 min. After methanol was removed, 0.1% CV in water was added to the biofilm and incubated for 10 min. Then CV-stained biofilms were washed with 1× PBS several times and dissolved in 2 mL of 95% ethanol. 200 μL of dissolved solution was transferred into another 96-well plate for spectroscopic reading at 570 nm.

On the other hand, the supernatant from the biofilms treated with compounds was diluted 10, 10^2^, 10^3^, 10^4^, 10^5^, and 10^6^-fold, and 20 μL of the diluent was streaked on MacConkey agar plate. The agar plates were incubated at 37 °C for 24 h, and the bacterial colonies were counted. The result was presented on a logarithmic scale. For biofilm-embedded bacterial cell viability assay, the treated biofilms fixed on the coverslips were dissolved in 2 mL of trypsin-EDTA solution in saline (1:10 = trypsin-EDTA: saline) and incubated for 15 min. 20 μL of the suspension from the well was diluted 10, 10^2^, 10^3^, 10^4^, 10^5^, and 10^6^-fold, and 20 μL of the diluent was streaked on MacConkey agar plate. The agar plate was incubated for 24 h and the bacterial colony was counted. The result was presented on a logarithmic scale.

### Resistance Development Study:

Ceftazidime, PCo, and the polymer-antibiotic combination were tested for resistance development against MDR *E. coli.* For the combination, the MIC values of ceftazidime were evaluated by keeping the polymer concentration constant at 64 μg mL^−1^. The first-passage MIC data of the selected combinations were obtained as described in the MIC study mentioned above. Then, bacteria cultured with the compound next to the last clear well were diluted to have 10^6^ CFU mL^−1^, which was used to conduct the second passage at 37 °C incubation for 18 h. By following the same steps, 6 subsequent passages were repeated.

### Statistical Analysis:

All acquired data were expressed as the mean ± SD (standard deviation), and sample size (*N*) for each statistical analysis in an experiment was given in the figure captions. GraphPad Prism 8 was used to conduct a one-way ANOVA analysis with Dunnett’s multiple comparison test, **** = *p* < 0.0001, *** = *p* < 0.001, ** = *p* < 0.01, * = *p* < 0.1, and ns = *p* > 0.1 (ns stands for nonsignificant). OriginPro was used for statistical analysis.

## Supplementary Material

Supporting Information

## Figures and Tables

**Table 1.** Antibacterial efficacy of ceftazidime in combination with PCo against *E. coli* (ATCC-25922), MDR *E. coli* (ATCC BAA-197), and *P. aeruginosa* (ATCC-27853).

Bacterial strains	MIC [μg mL^−1^]		Checkerboard assay			
	Ceftazidime	PCo	MIC, ceftazidime in combination	MIC, Polymer in combination	FIC Index	
					∑FIC	Activity
*E. coli*	0.25	256	0.25	8	1.03	A
			0.25	16	1.06	
			0.13	32	0.63	
			0.13	64	0.75	
MDR *E. coli*	64	512	16	8	0.27	S
			8	16	0.16	
			8	32	0.19	
			4	64	0.19	
*P. aeruginosa*	2	>512	1	8	<0.52	A
			1	16	<0.53	
			1	32	<0.56	
			0.5	64	<0.38	S

**Table 2.** Antibacterial efficacy of minocycline in combination with PCo against *E. coli* (ATCC-25922), MDR *E. coli* (ATCC BAA-197), and *P. aeruginosa* (ATCC-27853).

Bacterial strains	MIC [μg mL^−1^]		Checkerboard assay			
	Minocycline	PCo	MIC, minocycline in combination	MIC, Polymer in combination	FIC Index	
					∑FIC	Activity
*E. coli*	0.25	256	0.06	8	0.28	S
			0.06	16	0.31	
			0.06	32	0.38	
			0.03	64	0.38	
MDR *E. coli*	0.25	512	0.25	8	1.02	A
			0.25	16	1.03	
			0.13	32	0.56	
			0.13	64	0.63	
*P. aeruginosa*	8	>512	8	8	<1.02	A
			4	16	<0.53	
			2	32	<0.31	S
			1	64	<0.25	

**Table 3.** Antibacterial efficacy of rifampicin in combination with PCo against *E. coli* (ATCC-25922), MDR *E. coli* (ATCC BAA-197), and *P. aeruginosa* (ATCC-27853).

Bacterial strains	MIC [μg mL^−1^]		Checkerboard assay			
	Rifampicin	PCo	MIC, rifampicin in combination	MIC, Polymer in combination	FIC Index	
					∑FIC	Activity
*E. coli*	4	256	1	8	0.28	S
			0.25	16	0.13	
			0.25	32	0.19	
			0.25	64	0.31	
MDR *E. coli*	512	512	512	8	1.02	A
			512	16	1.03	
			256	32	0.56	
			256	64	0.63	
P. aeruginosa	16	>512	16	8	<1.02	A
			4	16	<0.28	S
			4	32	<0.31	
			2	64	<0.25	

## Data Availability

The data that support the findings of this study are available in the Supporting Information of this article.
