# Supplementary material for: Membrane-Active Metallopolymers: Repurposing and Rehabilitating Antibiotics to Gram-Negative Superbugs
Source: Adv Healthc Mater. Author manuscript; Available in PMC 2024 Feb 5. (PMC10842942; doi:10.1002/adhm.202301764)
Supplement: Supporting Information [file NIHMS1928924-supplement-Supporting_Information.pdf]

ADVANCED HEALTHCARE MATERIALS

Supporting Information

for *Adv. Healthcare Mater.*, DOI 10.1002/adhm.202301764

Membrane-Active Metallopolymers: Repurposing and Rehabilitating Antibiotics to Gram-Negative Superbugs

*JiHyeon Hwang, Swagatam Barman, Ruixuan Gao, Xiaoming Yang, Andrea O'Malley, Prakash Nagarkatti, Mitzi Nagarkatti, Maksymilian Chruszcz and Chuanbing Tang**

Membrane-Active Metallopolymers: Repurposing and Rehabilitating Antibiotics to Gram-negative Superbugs

JiHyeon Hwang[a], Swagatam Barman[a], Ruixuan Gao[b], Xiaoming Yang[c], Andrea O'Malley[a,d], Prakash Nagarkatti[c], Mitzi Nagarkatti[c], Maksymilian Chruszcz[a,d] and Chuanbing Tang[a]*

[a] Department of Chemistry and Biochemistry, University of South Carolina, Columbia, South Carolina 29208, United States

[b] Department of Chemistry, University of South Florida, Tampa, Florida 33620, United States

[c] Department of Pathology, Microbiology and Immunology, University of South Carolina, School of Medicine, Columbia, South Carolina 29209, United States

[d] Department of Biochemistry and Molecular Biology, Michigan State University, East Lansing, Michigan 48824, United States

*Corresponding Author Email: tang4@mailbox.sc.edu

Supporting Information

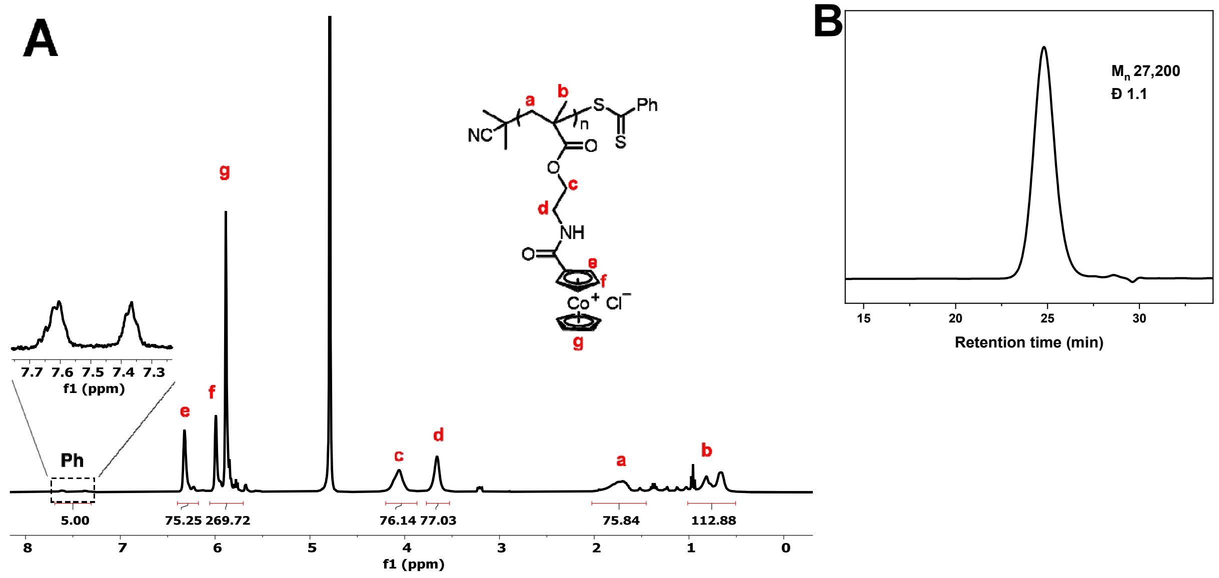

Figure S1. A) ^{1}H-NMR spectrum of PCo. The integration ratio of peaks at 7.3-7.7 ppm vs. 5.8-6.3 ppm is 5:345, indicative of a degree of polymerization at 38; B) GPC trace of PCo. Water GPC with pullulan standard polymer was employed for molecular weight calibration.

Table S1. MICs of PCo and ceftazidime against Gram-negative bacteria.

	E. coli	MDR *E. coli*	*P. aeruginosa*
PCo (μg/mL)	256	512	>512
Ceftazidime (μg/mL)	0.25	64	2

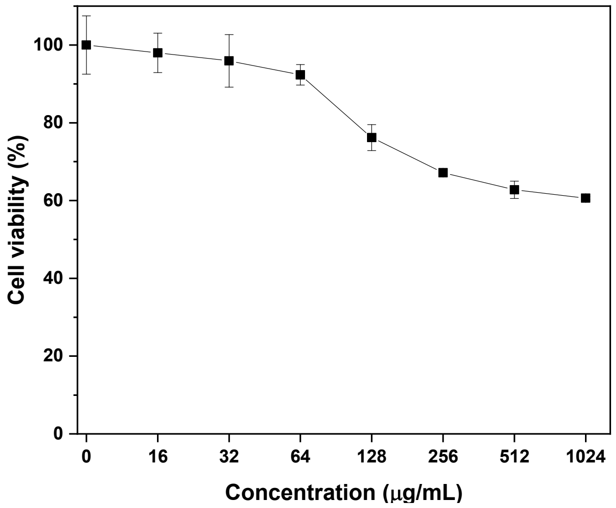

Figure S2. Cytotoxicity of HEK293T cells by PCo at increasing concentrations (16, 32, 64, 128, 256, 512, 1024 μg/mL).

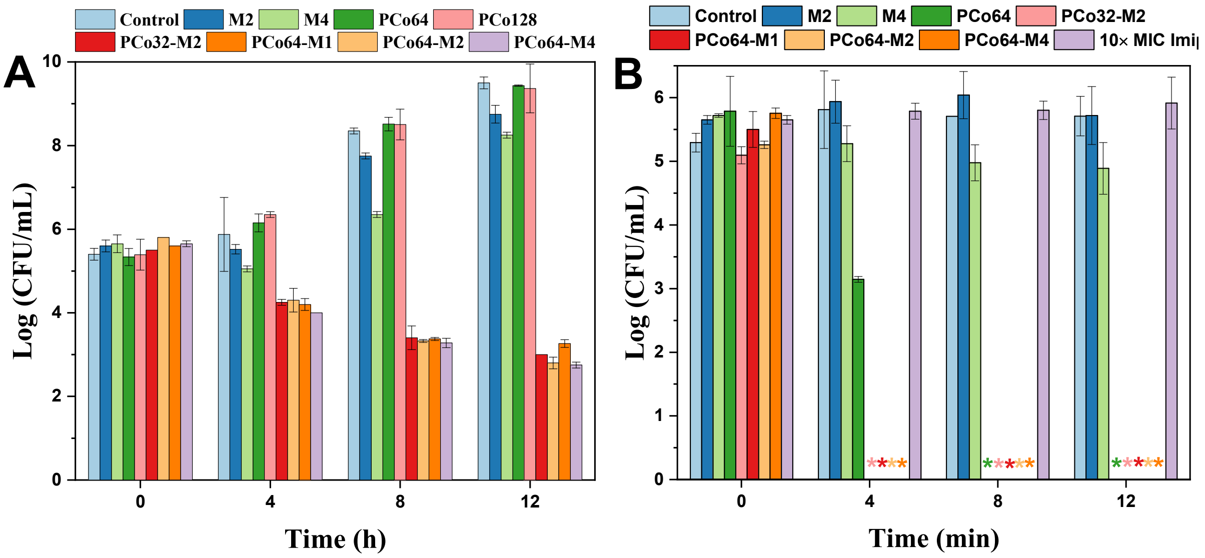

Figure S3. PCo, minocycline, and PCo-minocycline (PCo-M) combinations against A) *P. aeruginosa* and B) stationary phase of *P. aeruginosa*. 10 μg/mL of imipenem (10 × MIC) was used as a control. The asterisk (*) indicates <50 CFU. *N* = 3 for all studies.

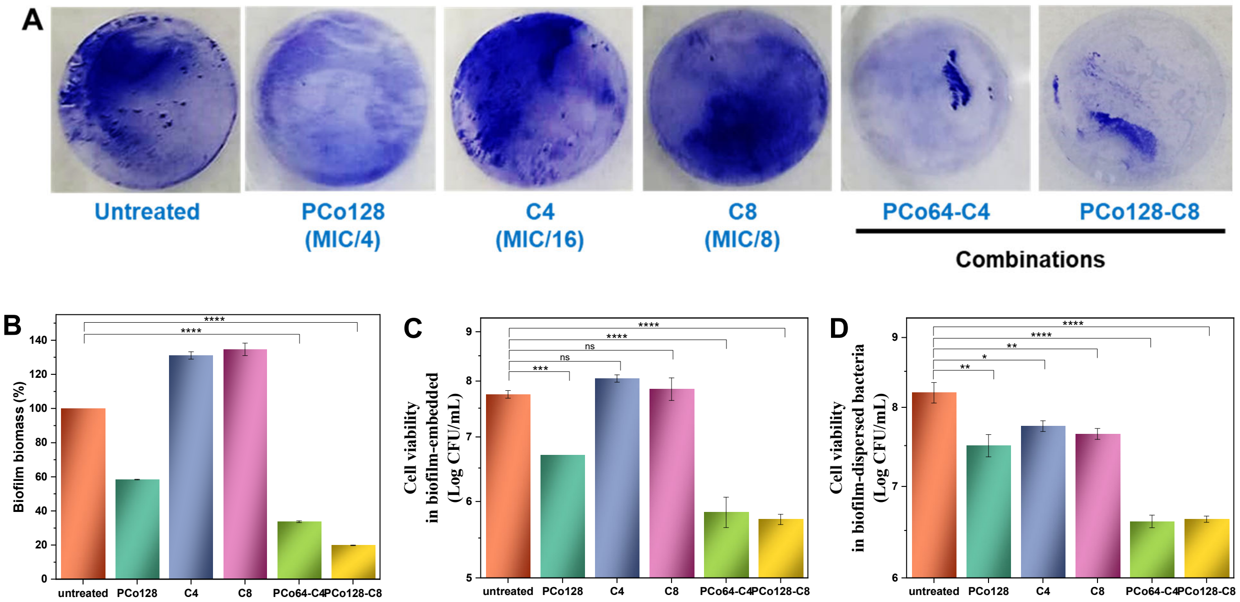

Figure S4. Biofilm disruption by PCo, ceftazidime and PCo-ceftazidime (PCo-C) combinations for MDR-*E. coli*: A) visualization by crystal violet staining; B) amount of biomass (%) in biofilms ($N = 4$); quantification of cell viability of C) biofilm-embedded bacteria ($N = 3$) and D) biofilm-dispersed bacteria ($N = 3$). ns, *, **, ***, and **** indicate $p > 0.1$, $p < 0.1$, $p < 0.01$, $p < 0.001$, and $p < 0.0001$, respectively, as determined by one-way ANOVA with Dunnett's multiple comparison test (ns stands for non-significant).
